# From Headache to Altered Consciousness: Headache Attributed to Hypoxia and/or Hypercapnia in a Smoker With Undiagnosed Chronic Obstructive Pulmonary Disease

**DOI:** 10.7759/cureus.71892

**Published:** 2024-10-19

**Authors:** Tatsuya Tanaka, Nao Furusho, Hirotoshi Kawashima, Tadasu Okaya, Akira Matsuno

**Affiliations:** 1 Department of Neurosurgery, International University of Health and Welfare Narita Hospital, Narita, JPN; 2 Department of Pulmonology, International University of Health and Welfare Narita Hospital, Narita, JPN; 3 Department of Allergy and Rheumatology, International University of Health and Welfare Narita Hospital, Narita, JPN

**Keywords:** altered consciousness, arterial blood gas analysis, chronic obstructive pulmonary disease, headache attributed to hypoxia and/or hypercapnia, hypercapnia, hypoxemia, magnetic resonance angiography, smoking

## Abstract

We present the case of a 57-year-old male with a history of smoking, hypertension, dyslipidemia, and migraines who experienced a one-month history of dyspnea and headaches, followed by sudden-onset altered consciousness. Initial imaging ruled out cerebrovascular and infectious etiologies, and arterial blood gas analysis revealed severe hypercapnia and hypoxemia (partial pressure of arterial carbon dioxide: 117 mmHg, partial pressure of arterial oxygen: 111 mmHg under a 10 L/min oxygen mask). The patient’s condition improved with mechanical ventilation, resulting in the resolution of both altered consciousness and headaches. Magnetic resonance angiography (MRA) demonstrated cerebral vasodilation, which normalized after treatment, confirming hypercapnia as the underlying cause. This case highlights the diagnostic challenges of differentiating headaches attributed to hypoxia and/or hypercapnia, particularly in smokers. MRA findings of cerebral vasodilation in the context of hypercapnia may provide a diagnostic clue.

## Introduction

The International Classification of Headache Disorders (ICHD) describes headaches occurring within 24 hours of acute hypoxemia, where the partial pressure of arterial oxygen (PaO₂) is below 70 mmHg, or in patients with chronic hypoxemia maintaining PaO₂ at or below this level [1,2]. Differentiating the contributions of hypoxemia and hypercapnia is often challenging, leading to classification as headache attributed to hypoxia and/or hypercapnia [1,2]. Both conditions can disrupt cerebral autoregulation, resulting in altered cerebral blood flow.

Hypercapnia induces vasodilation [3-5], potentially leading to headaches caused by cerebral hyperperfusion [6-8]. Chronic obstructive pulmonary disease (COPD) commonly results in hypercapnia, which may trigger headaches due to cerebral hyperperfusion.

Here, we report a case of a patient with a one-month history of dyspnea and headaches, progressing to sudden-onset altered consciousness, attributed to a headache attributed to hypoxia and/or hypercapnia.

## Case presentation

A 57-year-old male with a medical history of hypertension, dyslipidemia, and migraines presented with a one-month history of dyspnea and headaches, accompanied by fatigue and anorexia for two weeks. His headaches were exacerbated by smoking. He had been prescribed sodium valproate for his migraines and used sumatriptan as needed for headache relief. The patient had a 37-year smoking history (20 cigarettes per day) and consumed 60 grams of alcohol daily.

**Figure 1:**
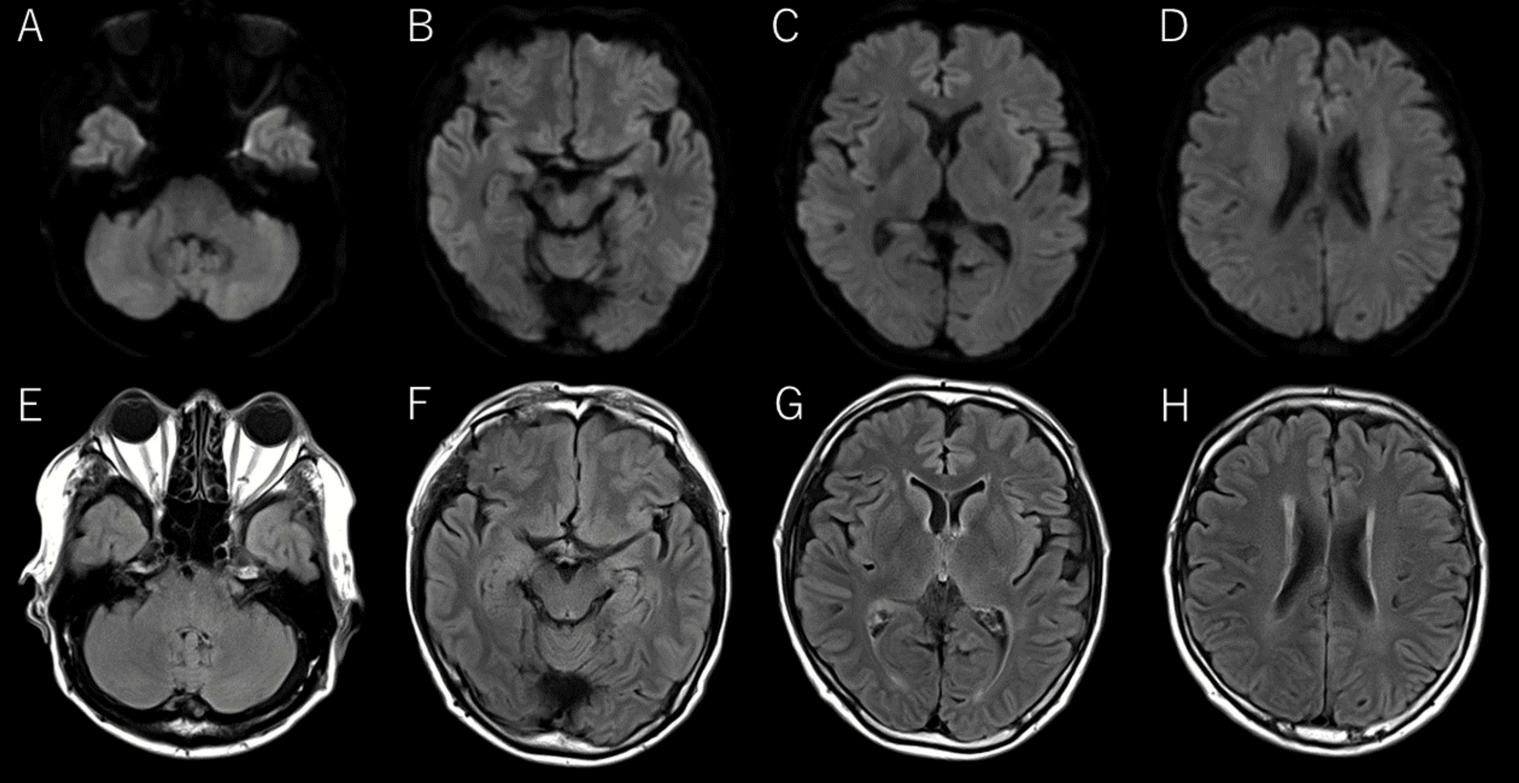
Initial brain imaging results The initial brain magnetic resonance imaging with diffusion-weighted imaging sequences (A-D) and fluid-attenuated inversion recovery sequences (E-H) showed no evidence of intracranial hemorrhage, brain tumor, or cerebral edema as a cause of the patient's headache.

**Figure 2:**
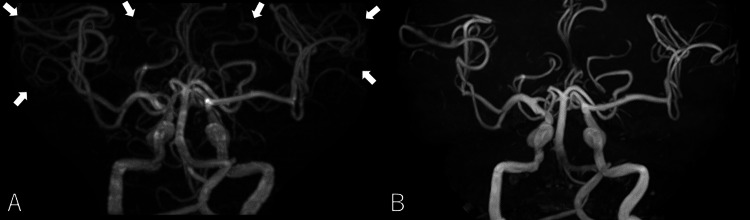
Magnetic resonance angiography findings (A) The initial magnetic resonance angiography (MRA) revealed no signs of arterial dissection or vasospasm, but significant vasodilation was observed (arrow). (B) On day 92, follow-up MRA demonstrated resolution of the previously noted vasodilation.

Initial diagnostic workup, including head computed tomography (CT), magnetic resonance imaging (MRI), magnetic resonance angiography (MRA), and cerebrospinal fluid analysis, excluded intracranial hemorrhage, venous sinus thrombosis, vascular dissection, and meningitis. MRA revealed cerebral vasodilation (Figures 1, 2A). The patient exhibited a Glasgow Coma Scale (GCS) score of 4 (E1V1M2) and a peripheral capillary oxygen saturation (SpO₂) of 68%. Bag-valve-mask ventilation at 10 L/min increased his GCS to 15 and SpO₂ to 100%. The patient was subsequently transferred to our emergency department.

Upon arrival at our emergency department, the patient’s GCS was 6 (E1V1M4), blood pressure was 197/109 mmHg, heart rate was 104 bpm, respiratory rate was 15 breaths per minute, and SpO₂ was 97% while receiving 10 L/min oxygen via a mask. Arterial blood gas analysis showed a pH of 7.163, partial pressure of arterial carbon dioxide (PaCO₂) of 117 mmHg, and PaO₂ of 111 mmHg. The patient was diagnosed with hypercapnia-induced altered consciousness. Endotracheal intubation and mechanical ventilation were initiated under analgesia, sedation, and muscle relaxation. Fursultiamine hydrochloride was administered due to a history of chronic alcohol consumption, and intravenous levetiracetam was administered for suspected status epilepticus.

**Figure 3:**
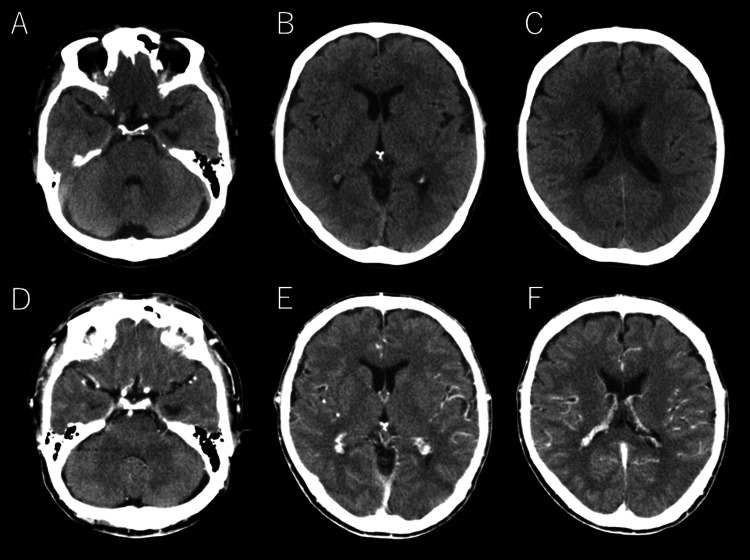
Head computed tomography results at admission Non-contrast head computed tomography (CT) (A-C) and contrast-enhanced CT (D-F) showed no evidence of intracranial hemorrhage or venous sinus thrombosis as the cause of the patient's headache.

Repeat head CT showed no hemorrhage, and contrast-enhanced CT excluded arterial dissection and venous thrombosis (Figure 3).

**Figure 4:**
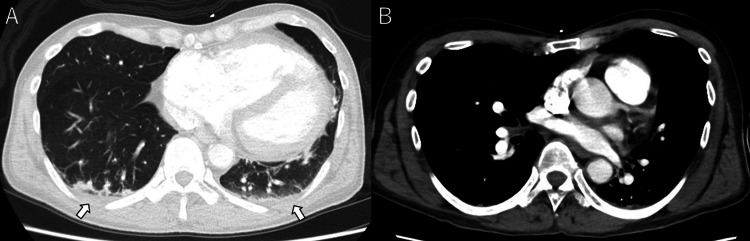
Chest computed tomography findings at admission (A) Non-contrast chest computed tomography (CT) revealed bilateral dorsal infiltrates, suggestive of pneumonia (arrow). (B) Contrast-enhanced chest CT showed no detectable thrombus in the pulmonary arteries.

Chest and abdominal CT ruled out pneumothorax and pulmonary embolism (Figure 4).

After the correction of hypercapnia, the patient’s GCS returned to 15, and his altered consciousness resolved. He was weaned off the ventilator on the third hospital day, and his headaches also subsided. Inhaled short-acting beta-agonists and 60 mg of prednisolone were administered for hypercapnia and hypoxemia due to COPD. Treatment was transitioned to inhaled long-acting muscarinic antagonists. Pulmonary function testing revealed a forced expiratory volume in one second of 65.5%, confirming a diagnosis of COPD, which was identified as the underlying cause of his headaches and altered consciousness. The patient was discharged after 15 days, having quit smoking, and has reported no recurrence of headaches. A follow-up MRI three months later showed resolution of cerebral vasodilation on MRA (Figure 2).

## Discussion

This case describes a patient with hypercapnia-induced headaches, which subsequently progressed to a disturbance of consciousness. Headache attributed to hypoxia and/or hypercapnia occurs in the context of decreased oxygen or elevated carbon dioxide levels [1,2]. According to the ICHD-2 criteria, distinguishing the effects of hypoxemia from hypercapnia is often challenging [1]. The ICHD-2 defines hypoxemia-induced headaches as those occurring within 24 hours of acute hypoxemia, where PaO_2_ is less than 70 mmHg, or in patients with chronic hypoxemia, where PaO_2_ remains below this threshold [1]. In ICHD-3, the diagnosis is confirmed when the headache occurs in close temporal association with the exposure and there is evidence of a causal relationship. This is demonstrated by a significant worsening of the headache as hypoxemia or hypercapnia worsens or by an improvement in symptoms following the resolution of these conditions. Additionally, the headache must not be attributable to any other diagnosis outlined in the ICHD-3 criteria [2]. Conditions associated with acute or chronic hypoxemia and/or hypercapnia, such as pulmonary diseases (e.g., asthma, COPD), cardiac conditions (e.g., congestive heart failure), or hematologic abnormalities (e.g., severe anemia), may precipitate such headaches. In this case, a patient with undiagnosed COPD developed headaches triggered by hypoxia and hypercapnia related to smoking. The symptoms resolved as hypoxia and hypercapnia improved. The patient met the ICHD-3 criteria, supporting the diagnosis of a headache attributed to hypoxia and/or hypercapnia.

Initially, this patient's headaches were misdiagnosed as migraines and treated accordingly. In patients with migraines, smoking and the use of heated tobacco products can act as headache triggers [9,10]. Moreover, smoking and obesity are well-known risk factors for medication-overuse headaches [9,10]. Chronic hypoxemia is common in smokers. Pulse oximetry cannot differentiate between carboxyhemoglobin and oxyhemoglobin, leading to falsely elevated oxygen saturation readings in smokers [11]. Without arterial blood gas analysis, hypoxemia and hypercapnia may go undetected. Therefore, in smokers, headache attributed to hypoxia and/or hypercapnia should be considered in the differential diagnosis alongside migraines. The prevalence of headache attributed to hypoxia and/or hypercapnia in smokers is currently unknown, warranting further investigation.

Cerebral autoregulation is influenced by both hypoxemia and hypercapnia, which can alter cerebral blood flow [3-5]. In this case, MRA revealed cerebral vasodilation during headache evaluation. CO₂, a potent cerebral vasodilator, induces vasodilation by reducing intracellular free calcium and activating adenosine triphosphate-sensitive potassium ion channels, which cause smooth muscle cell hyperpolarization [4]. The cerebral vasodilation observed on MRA was likely due to hypercapnia. In cases where headaches are accompanied by altered consciousness and widespread vasodilation is observed on MRA, hypercapnia should be considered in the diagnostic workup.

## Conclusions

This case highlights the critical importance of including hypoxemia and hypercapnia in the differential diagnosis of headaches, particularly in patients with underlying respiratory disorders, such as smokers. Cerebral vasodilation, as detected on MRA, may serve as a key diagnostic marker for hypercapnia-induced cerebral dysfunction. Prompt recognition and correction of hypercapnia are essential to prevent further neurological deterioration. Recognizing this association can facilitate early diagnosis and targeted treatment, ultimately improving clinical outcomes.
